# Understanding patient acceptance and refusal of HIV testing in the emergency department

**DOI:** 10.1186/1471-2458-12-3

**Published:** 2012-01-03

**Authors:** Katerina A Christopoulos, Sheri D Weiser, Kimberly A Koester, Janet J Myers, Douglas AE White, Beth Kaplan, Stephen F Morin

**Affiliations:** 1 San Francisco General Hospital HIV/AIDS Division, University of California San Francisco, San Francisco, CA, USA; 2 Center for AIDS Prevention Studies, University of California San Francisco, San Francisco, CA, USA; 3 Department of Emergency Medicine, Alameda County Medical Center, Highland Hospital, Oakland, CA. USA; 4 Department of Emergency Medicine, San Francisco General Hospital, University of California San Francisco, San Francisco, CA, USA

**Keywords:** Emergency department, HIV testing, HIV test refusal, HIV test acceptance

## Abstract

**Background:**

Despite high rates of patient satisfaction with emergency department (ED) HIV testing, acceptance varies widely. It is thought that patients who decline may be at higher risk for HIV infection, thus we sought to better understand patient acceptance and refusal of ED HIV testing.

**Methods:**

In-depth interviews with fifty ED patients (28 accepters and 22 decliners of HIV testing) in three ED HIV testing programs that serve vulnerable urban populations in northern California.

**Results:**

Many factors influenced the decision to accept ED HIV testing, including curiosity, reassurance of negative status, convenience, and opportunity. Similarly, a number of factors influenced the decision to decline HIV testing, including having been tested recently, the perception of being at low risk for HIV infection due to monogamy, abstinence or condom use, and wanting to focus on the medical reason for the ED visit. Both accepters and decliners viewed ED HIV testing favorably and nearly all participants felt comfortable with the testing experience, including the absence of counseling. While many participants who declined an ED HIV test had logical reasons, some participants also made clear that they would prefer not to know their HIV status rather than face psychosocial consequences such as loss of trust in a relationship or disclosure of status in hospital or public health records.

**Conclusions:**

Testing for HIV in the ED as for any other health problem reduces barriers to testing for some but not all patients. Patients who decline ED HIV testing may have rational reasons, but there are some patients who avoid HIV testing because of psychosocial ramifications. While ED HIV testing is generally acceptable, more targeted approaches to testing are necessary for this subgroup.

## Background

To facilitate earlier detection of HIV infection, the Centers for Disease Control and Prevention (CDC) issued guidelines in 2006 recommending routine HIV screening of all adults ages 13-64 in all health care settings, including the emergency department (ED) [1]. Since the release of these guidelines, ED HIV testing has been shown to be feasible and acceptable across a spectrum of consent, testing, and patient selection strategies [2-5]. Patient satisfaction rates with ED HIV testing range from 80-90% [6-11].

Despite these favorable attitudes, the proportion of patients who accept ED HIV testing varies widely, ranging from 24%-91% [8,12]. Demographic factors associated with refusal of HIV testing are older age, white race, female sex, higher income, and being married [12,13]. Common reasons for refusal are having been tested recently, feeling sick, and the perception of being at low risk for HIV infection [5,7].

A current focus of research is how patients' perceived and actual HIV risk influences decisions around HIV testing, as it is thought that patients who decline ED HIV testing may be at greater risk for HIV infection. A study in a Boston ED found that 15% of subjects who perceived a need for testing based on their risk for HIV infection ultimately refused testing [13]. In Washington D.C., researchers tested de-identified blood samples from decliners and found nearly three times the risk of HIV infection compared to patients who accepted testing [14].

The potential importance of testing individuals who decline ED HIV testing calls for more insight into how patients make decisions around testing. There are many quantitative assessments of ED HIV testing uptake, but to date there has been no detailed exploration of why people accept or decline HIV testing in the ED. Thus, the objective of this study was to use in-depth interviews to develop a better understanding of acceptance and refusal of ED HIV testing.

## Methods

We conducted in-depth interviews with in fifty patients (28 who accepted HIV testing and 22 who declined HIV testing) three northern California EDs. The ED settings have been described in detail elsewhere [15]. All sites were recipients of CDC funding to increase HIV testing among disadvantaged urban populations. As previously described, [15] two sites in Oakland offered non-targeted oral swab screening at registration or triage while a third site in San Francisco utilized clinician-initiated diagnostic venipuncture testing and targeted testing of admitted patients [16].

All patients offered an HIV test in the ED were eligible to participate in this study, although recruitment of patients was limited to those who had already received their test results. Patients with reactive test results were excluded from recruitment given the sensitive nature of these test results and the need for immediate follow-up counseling. In addition, to gain a range of perspectives, study investigators decided *a priori *to attempt to sample ten accepters and ten decliners at each site. Emergency department staff helped identify patients for recruitment, and flyers advertising the study were posted throughout the EDs. Once patients were recruited, researchers explained the goals of the study and obtained verbal consent for an interview. Interviews were conducted in a private setting in the ED and lasted approximately twenty to thirty minutes. Information on demographics and health care utilization, including age, gender, race/ethnicity, insurance/care status, and reason for ED visit, was collected at the beginning of each interview. Participants were each reimbursed thirty dollars for their participation. Study data were collected from April to June 2009. The institutional review boards of the University of California San Francisco and all participating sites approved this study.

A semi-structured interview guide was developed to cover participant experiences with HIV testing in the ED, reasons to accept or decline HIV testing, prior HIV testing history, relationship status and perceptions of HIV risk behavior, and participant attitudes towards HIV infection. Interviews were transcribed verbatim and entered into Atlas.ti [17] for organization and easy retrieval of text elements. Three analysts employed a framework analysis approach to the data, which included two distinct analytic tasks: managing and interpreting the data [18]. The analysts began managing the data by reading a subset of the interviews to gain an initial familiarity with the dataset and to produce a preliminary list of coding categories. Subsequent steps included refinement of codes, as well as sharpening the shared understanding of how to apply codes. Each interview was coded by a primary analyst and reviewed by a secondary analyst. The analytic team then selected text associated with key codes across cases to be read and summarized as a group in six three-hour interpretative analysis sessions. The goal during the interpretative phase was to produce an exploratory analysis to uncover overarching attitudes towards HIV testing in the context of a visit to the ED.

## Results

### Respondent Characteristics

Participants represented a diverse range of ages and were evenly divided between men and women (Table 1). Reflecting the demographics of the communities these EDs serve, half of the participants in this study were African-American. While we purposively sampled to obtain an equal number of accepters and decliners of HIV testing, it was more difficult to recruit decliners at Site 1, which used clinician-initiated testing, than at sites 2 and 3, where testing was offered at a central location (e.g. registration, triage). It is not clear whether this was because fewer patients declined clinician-initiated testing or because ED clinicians were simply too busy to refer decliners to the study. Thus, the research team stopped recruitment at site 1 once the quota of accepters and decliners was reached at the other sites (Table 2). About half of participants had insurance and a regular source of medical care. The majority of patients (44/50, 88%) had previously tested for HIV infection, including 18 (82%) of the 22 decliners sampled. Of the 6 participants who had never tested for HIV infection, 2 decided to accept testing in the ED that day.

**Table 1:** Respondent Characteristics (n = 50)

Age Category	
18-29 years	17 (34%)
30-49 years	19 (38%)
> = 50 years	14 (28%)
Gender	
Male	26 (52%)
Female	24 (48%)
Race/Ethnicity	
African-American	26 (52%)
White	11 (22%)
Latino	3 (6%)
Asian	3 (6%)
Mixed Race	5 (10%)
Other/Unknown	2 (4%)
Accepters of HIV Testing	
Yes	28 (56%)
No	22 (44%)
Prior HIV Testing	
Yes	43 (86%)
No	6 (12%)
Not Sure/Unknown	1 (2%)
Have Insurance	
Yes	26 (52%)
No	20 (40%)
Not Sure/Unknown	4 (8%)
Have Regular Source of Care	
Yes	22 (44%)
No	27 (54%)
Not Sure/Unknown	1 (2%)
Reason for ED Visit (as reported to interviewer)	
Pain	15 (30%)
Trauma	14 (28%)
Infection/Cough/Fever	7 (14%)
Dermatologic	5 (10%)
Possible HIV Exposure	4 (8%)
Hyperglycemia	3 (6%)
Out of Medication	1 (2%)
Ear Irrigation	1 (2%)

**Table 2:** Acceptance and Refusal by Site

Site	Accepted	Refused	Total
Site 1: Clinician-Initiated Diagnostic/Targeted Testing Using Venipuncture Specimens	8	1	9

Site 2: Opt-in Non-Targeted Oral Swab Testing Offered at Triage	10	11	21

Site 3: Opt-out Non-Targeted Oral Swab Testing at Registration	10	10	20

Total	28	22	50

### Reasons for Acceptance

The most common reasons for accepting HIV testing in the ED were because participants "just wanted to know" and that it was "good to know" one's HIV status (Table 3). Participants viewed the test as a form of assurance of negative status. Some participants saw HIV testing as a way to "check" their bodies and ensure good health.

**Table 3:** Reasons for Acceptance or Refusal of HIV Testing in the ED

Reasons for Acceptance	Reasons for Refusal
Curiosity/Assurance	Recent Negative Test
Convenience/Opportunity	Perception of Being at Low Risk
Perception of Being at Risk	Feel Sick/Only Want to Address Reason for ED Visit
Perception of Being at Low Risk	Don't Want to Know
Encouraged by Partner	Confidentiality Concern
Ensure Safety of Others	Potential Strain on Relationship
Testing for HIV As Any Other Health Problem Free	Prefer to Test with Primary Care Physician

Well, I'm in here getting tested. I'm getting my liver tested, my kidneys tested. I had an ultrasound on the veins in my leg, so why not get an HIV test. They already have the blood, so why not?

-51-year-old African-American man

One participant described how an HIV test in a medical setting could help her health:

The hospital ask you anything about your health that they damn well want to. If it's helping, ask me.

-42-year-old African-American woman

Participants emphasized HIV testing in the ED as an opportunity they might not have otherwise encountered or sought out, and they perceived their acceptance of testing as taking advantage of that opportunity. Convenience and the fact that tests were free were also cited as reasons to accept testing.

Because I'd never had one and it was free... I would have not sought out at test just on my own. I wouldn't have made a doctor's appointment. So it was a good thing that the hospital offered it. I went, "Well, why not?" But I wouldn't have thought to go, 'cause it wasn't a concern in my mind, whether that's right or wrong smart or ignorant. I think it's a good thing because everybody should know and it's one less thing you have to worry about.

-52-year-old White woman

One participant who had never taken an HIV test liked having the test as part of her medical care in the ED. She acknowledged that stigma played a role in her decision to test in the ED as opposed to a dedicated testing center.

Actually - to be honest - I think that I would never have taken a test if it wasn't offered at this hospital. Because for me to go to a clinic that does HIV testing - it would make me really uncomfortable. Because just the idea of having it stated boldly and well known as an HIV testing center - for me to show my face in an area like that - I would feel a little uncomfortable and just in case I might see someone I know I would feel as if "Well, this person might think I have something and they might go around spreading rumors and say, 'Hey I saw her at the HIV testing center. She might have something.'" So it would make me uncomfortable but I actually took a test here because it was very convenient. I was like, "Wait a minute - maybe I do want to know." And I guess it is a little more discreet so I kind of like that better than having to go to a clinic that everyone knows is testing for HIV.

-25-year-old Asian woman

Similarly, another participant appreciated the absence of a traditional counseling approach.

I had no intention of getting an HIV test when I came in here so it was just added on to what my purpose was being here. I felt pretty confident I didn't have HIV. I felt pretty confident the first time around too but the first time around (testing) was done with a lot more intention on my part and there was counseling involved, so it was a much more elaborate experience that this was. I didn't express any interest in counseling. I think had they gone that route I probably wouldn't have wanted to take it because I'm not here for that so it was sort of like, "Oh well, while you're here..." And the fact that they presented it as "while you're here, by the way..." I actually thought was pretty good. Because it made me feel comfortable. It wasn't like, "Oh, my God, you have to make sure everybody who comes through here doesn't have AIDS." Their casual attitude for me was fine. It worked out very well.

-55-year-old White woman

Finally, other reasons for acceptance were that some participants felt they had engaged in behavior that put them at risk for acquiring HIV infection, they had partners who encouraged them to test, and they wanted to ensure the safety of others as they entered new relationships.

### Reasons for Refusal

Many participants declined HIV testing because they had tested recently, often in the same ED testing program (Table 3). In addition, individuals did not perceive themselves to be at risk for HIV infection, usually because they were in long-term monogamous relationships and had been tested prior to or during these relationships.

I took one during my last pregnancy; I just have a 4-month-old at home so I'm monogamous and I didn't see any need to waste the tester.

-30-year-old mixed-race woman

Other individuals who did not perceive themselves at risk for HIV infection stated that they had been abstinent or used protection consistently since their last HIV test.

Cause I don't got it. I tested and I had a blood test and a swab test and it was in '06 and this is '09. But since I was safe, I was in custody when they did it, tested it and since I've been home I've been using straight condoms and I don't kiss nobody with sores in their mouths, stuff like that.

-38-year-old African-American man

In addition, a few participants alluded to wanting to focus on the medical issue that brought them to the ED, even though they may have tested for HIV in the past.

'Cause I just came here for my toe. I didn't want to do nothing else.

-27-year-old Hispanic woman

While nearly all participants had favorable views on testing for HIV infection in the ED, there was case where a participant expressed a desire for a more nuanced conversation within the bounds of an established patient-provider relationship. She described her rationale for declining the test as follows:

One, it's because this is the county hospital. The approach of the person who asked me was a little raunchy, like, "Hey, you want to take an HIV?" No information or nothing like that. I feel like I have the right to know. I think I know my status and I pray it hasn't changed since I've known it and I would just feel more comfortable at my regular physician, you know what mean, far as if there was something I needed to consult them about or something of that nature; just the confinements of the relationship that I've already established with my current provider. I would just feel more comfortable and for them having a record or whatever.

-25-year-old African-American woman

Of the four participants who declined HIV testing and had never previously tested for HIV infection, two participants stated that they felt at low risk for HIV infection because of being in long-term monogamous relationships. Another participant stated, "I don't know, actually. I just don't want to take one. I guess I don't want to know anything." While this participant did not feel that HIV testing was necessary for him at the moment because he had not had sex in eighteen months, he did go on to endorse HIV testing as important in general. Another patient gave a layered response, explaining that she perceived herself at low risk because of being in a monogamous relationship, but she also voiced concerns about the potential disruption of trust in that relationship and the confidentiality of test results if she was found to be HIV-infected. She also expressed not wanting to know her HIV status.

Um, because I have been with the same guy for more than 20 years and I'm not having any outside sex so I just - there's really no privacy once you get that information out there... I don't care to know one way or the other... I think my risk is low, I mean I'm trusting him not to be having any outside sex and I know that I'm not and like I said we've been in a monogamous relationship for more than twenty years now so I don't think that I'm at risk, no... Should I start a medical record somewhere I don't want that I was even tested because really supposed confidentiality is not reality. Everything's on computer. I just don't want that information out there. Um, well I would have to be tested so there my confidentiality is violated cause if it's positive that's everybody. Public health, the lab, everybody would know. And I don't want to know. Like I said - well I guess that would ruin whatever trust, cause I'd know where I got it from if I got it. Like I said I haven't had sex with anybody but him so I just prefer not to know.

-50-year-old African-American woman

Overall, many of the decliners had personal experiences with HIV, including family members and friends who died of AIDS. Decliners were more likely to discuss HIV stigma compared to the accepters, including descriptions of "layered" stigma around homosexuality and intravenous drug use [19]. One participant who declined HIV testing and had never tested for HIV infection described a family's response to a cousin who died of AIDS.

And I remember one year Christmas dinner, Thanksgiving dinner, we used to get together to have a kind of potluck thing at different people's houses - my grandmother or one of my aunts. And he wanted to come to dinner. That was fine, but he wanted to bring his partner and like hell no. No, you can't bring him. They fixed him a plate and told him to take it with him. Don't worry about bringing it back.

-50-year-old African-American woman

It is worth noting that no participant invoked test type (oral swab vs. venipuncture) as a reason for declining the test.

### The Experience of Testing for HIV in the ED

All participants described being given the opportunity to decline HIV testing in the ED, and nearly all participants were satisfied with the offer of HIV testing. As most of the people who accepted testing had tested for HIV infection previously, they acknowledged having familiarity with HIV testing. Only one person expressed a desire for more counseling.

It's kind of like a rush here, so they don't really sit down and really talk to you about that test and they just want to test you for when you do come back and you already been on record as negative or positive or whatever... I wish they could talk to me about the test and everything like that but they don't because they be so backed up to the point where they can't talk to you and they just be like, "Okay, well here go the test. Your doctor's going to tell you the results." But it would be nice you know if they would give a little background and tell them how the test is and how you can just get it far as even having sex transmitted, just being sexual with your partner or you can get it from kissing or whatever like somebody bleed or somebody get cut and you try to help them clean up and they might be having it and your blood touch their blood and damn you got it.

-19-year-old African-American woman

In contrast to most participants, this respondent felt she was at high risk for HIV infection because in the past she had an HIV-infected partner. She wanted the opportunity to speak with someone about her situation and also wanted more information on risks of HIV transmission. Her story was the exception in this dataset, but it demonstrates that those at increased risk of HIV infection may continue to benefit from counseling at the time of testing.

## Discussion

Participants described a number of factors that influenced their decision to accept testing, including curiosity, reassurance of negative status, convenience, and opportunity. Bringing the test to patients in the ED removed logistical and psychological barriers that are known to prevent people from seeking out testing in traditional venues [20]. In addition, treating HIV like any other health problem helped patients feel comfortable about the HIV testing process. With regard to refusal of HIV testing in the ED, we found that reasons were having been tested recently and wanting to focus on the medical issue that brought the patient to the ED, consistent with other studies [5,7]. Other reasons were "not wanting to know" and fear of confidentiality violations. The role of patients' perception of HIV risk in testing decisions was more complex. The perception of being at risk for HIV infection was certainly a motivation to accept testing, as at voluntary and counseling testing sites [21], however, we found that the perception of being at low risk for HIV infection was a reason for both refusal and acceptance, as it allowed individuals to feel comfortable accepting a test that they may not have sought elsewhere.

We discovered that many decliners provided logical reasons for refusing the test. Even so, decliners viewed HIV testing in general as important and interpreted the offer to test as an expression of concern on the part of the medical establishment. However, our data suggest that even patients who support HIV testing and are aware of its benefits may choose not to test because they prefer to live in uncertainty rather than face psycho-social consequences such as partner discord or discrimination based on HIV status. Indeed, the decliners in our study were more likely to describe instances of HIV stigma, even though there was no conscious acknowledgment of HIV stigma in the decision-making process.

There are several limitations to this study. This qualitative data is hypothesis-generating rather than definitive, and it may not be generalizable to other ED HIV testing programs. In addition, the interviews were done in busy EDs with patients who had pressing medical issues, thus participants may not have been as reflective as they would have been in other settings. Since the goal of this investigation was to look across rather than within programs, we did not assess how operational aspects of the three different models of ED HIV testing may have affected acceptance or refusal of testing. We were only able to recruit one decliner from the site that used clinician-initiated testing, as referrals of decliners at that site had to come directly from ED clinicians who had multiple competing priorities and may have been too busy to refer patients to the study. Finally, we did not systematically ascertain when patients last tested, since at the time of this study, these programs did not have policies on repeat testing. To our knowledge, there are no published guidelines on repeat HIV testing in the ED. In general, the 2006 CDC guidelines suggest at least annual testing of high-risk individuals with repeat testing of other individuals based on clinical judgment [1]. Thus, it is important to acknowledge that repeat testing may not have been necessary for some of the individuals who cited recent testing as a reason for refusal. Indeed, the optimal interval for repeating an HIV test in the ED is an important area of future research.

## Conclusions

Participants in this study appreciated HIV testing as part of their ED care and for the most part did not feel the need for counseling with testing. Offering testing for HIV as for any other health problem facilitated acceptance for many participants. For several participants, this type of offer was not compelling enough, and they did not necessarily perceive HIV testing as normative. In order to reach this group, some studies have suggested increasing education about the rationale and benefits of testing [13]. However, the results of this study demonstrate that education alone may not address concerns that are related to potential psychosocial consequences of testing and that these concerns may supersede a patient's willingness to receive screening tests that benefit overall health. While acknowledging that all patients have the right to refuse testing at any time and for any reason, further research is needed to better understand these concerns and develop interventions to address them. It is likely that these interventions will require more counseling than is currently available in ED HIV testing programs, thus assessment of feasibility will be a key consideration in moving this research agenda forward.

## Competing interests

The authors declare that they have no competing interests.

## Authors' contributions

SW, JM, and SM conceived the study and obtained research funding. DW and BK helped recruit participants. KC, KK, and SW analyzed the data. KC drafted the manuscript and all authors contributed substantially to its revision. All authors read and approved the final manuscript.

## Pre-publication history

The pre-publication history for this paper can be accessed here:

http://www.biomedcentral.com/1471-2458/12/3/prepub
